# Putting the BRK on breast cancer: From molecular target to therapeutics

**DOI:** 10.7150/thno.49716

**Published:** 2021-01-01

**Authors:** Hui Li Ang, Yi Yuan, Xianning Lai, Tuan Zea Tan, Lingzhi Wang, Benjamin BoJun Huang, Vijay Pandey, Ruby Yun-Ju Huang, Peter E. Lobie, Boon Cher Goh, Gautam Sethi, Celestial T. Yap, Ching Wan Chan, Soo Chin Lee, Alan Prem Kumar

**Affiliations:** 1 Cancer Science Institute of Singapore and Department of Pharmacology, Yong Loo Lin School of Medicine, National University of Singapore, Singapore; 2 Cancer Science Institute of Singapore, National University of Singapore, Singapore.; 3 Tsinghua Berkeley Shenzhen Institute and Division of Life Science and Health, Tsinghua University Graduate School, Shenzhen, China.; 4 Shenzhen Bay Laboratory, Shenzhen, Guangdong Province, China.; 5 School of Medicine, College of Medicine, National Taiwan University, Taipei, Taiwan.; 6 Department of Haematology-Oncology, National University Health System, Singapore.; 7 National University Cancer Institute, National University Health System, Singapore.; 8 Department of Pharmacology, Yong Loo Lin School of Medicine, National University of Singapore, Singapore.; 9 Department of Physiology, Yong Loo Lin School of Medicine, National University of Singapore, Singapore.; 10 Department of Surgery, University Surgical Cluster, National University Hospital, Singapore.

**Keywords:** Breast tumor kinase (BRK), hallmarks of cancer, chemical inhibitors, molecular inhibitors, meta-analysis

## Abstract

BReast tumor Kinase (BRK, also known as PTK6) is a non-receptor tyrosine kinase that is highly expressed in breast carcinomas while having low expression in the normal mammary gland, which hints at the oncogenic nature of this kinase in breast cancer. In the past twenty-six years since the discovery of BRK, an increasing number of studies have strived to understand the cellular roles of BRK in breast cancer. Since then, BRK has been found both *in vitro* and *in vivo* to activate a multitude of oncoproteins to promote cell proliferation, metastasis, and cancer development. The compelling evidence concerning the oncogenic roles of BRK has also led, since then, to the rapid and exponential development of inhibitors against BRK. This review highlights recent advances in BRK biology in contributing to the “hallmarks of cancer”, as well as BRK's therapeutic significance. Importantly, this review consolidates all known inhibitors of BRK activity and highlights the connection between drug action and BRK-mediated effects. Despite the volume of inhibitors designed against BRK, none have progressed into clinical phase. Understanding the successes and challenges of these inhibitor developments are crucial for the future improvements of new inhibitors that can be clinically relevant.

## Introduction

**Figure 1:**
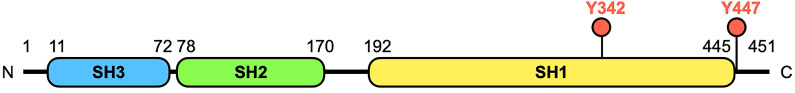
** Structure and domains of BRK.** The human BRK protein is a 451 amino acid kinase, which consists of 3 functional domains - SH3, SH2, and SH1 domain. The first two domains are required for interactions with other molecules, while the SH1 domain confers a catalytic role to the protein. Tyrosine 342 (Y342) is a phosphorylation site in the tyrosine kinase (TK) domain that increases BRK's activity, while tyrosine 447 (Y447) phosphorylation negatively regulates kinase activity 6, 11.

Breast tumor kinase (BRK), also known as protein tyrosine kinase 6 (PTK6), was originally cloned from a metastatic human breast tumor in 1994 1. The BRK transcript is encoded by an 8.93 kb length DNA located on chromosome 20q13.3 in humans 2. The protein is a 451-amino acid kinase, comprising of 3 parts, a classic Src homology 3 (SH3) domain and a Src homology 2 (SH2) domain, both of which are involved in protein-protein interactions, and a tyrosine kinase (SH1) domain (**Figure 1**). However, compared to members of the Src family, BRK lacks an amino-terminal myristoylation sequence, which makes this protein soluble and accessible for interactions with intracellular substrates.

Since its discovery, there have been a growing number of publications on the expression levels and functions of this intracellular, non-receptor tyrosine kinase in various cell types, particularly in breast cancer. BRK is highly expressed in tumor samples of the breast, ovary, and cervix, while its expression is low to undetectable in normal tissues 3-5. More importantly, studies have shown that BRK overexpression is associated with poorer overall survival in breast cancer 6, 7.

Apart from its overexpression in cancer, there is evidence that subcellular localization of BRK can also contribute to its oncogenic function. BRK was found to be localized to the cytosol in breast cancers 8. It was discovered through immunofluorescence and immunohistochemistry staining that that the low levels of BRK found in normal mammary tissues were nuclear targeted, whereas the BRK found in transformed cells was plasma membrane-localized, with BRK becoming more cytoplasmic in higher grade tumors 6.

In addition to cellular localization, the phosphorylation status of two tyrosine residues, Y342 and Y447, on BRK is also associated to oncogenicity (**Figure 1**). Peng *et al.* provided an additional dimension to the understanding of BRK in cancer when they discovered that there is no phosphorylation of Y342 in normal mammary tissues, in contrast to breast cancer tissues 6. Similarly, in ovarian and prostate cancer, protein tyrosine phosphatase 1B (PTP1B) and phosphatase and tensin homolog (PTEN) respectively were found to dephosphorylate BRK on Y342 to inhibit BRK activity 9, 10. On the other hand, phosphorylation at site Y447 negatively regulates kinase activity 11.

In cancer development, cells progressively acquire damage that leads to its eventual transformation into cancer cells. These damage markers are classically termed by Hanahan and Weinberg to be “Hallmarks of Cancer” 12, 13. Due to its ubiquitous nature, BRK was discovered to affect a large number of pathways, which contribute to various hallmarks of cancer. To date, most research that has been done on BRK heavily focuses on BRK's involvement in breast cancer. We therefore seek to provide a comprehensive review on BRK in breast cancer, particularly its contribution to each hallmark of cancer. Given the oncogenic role of BRK, we present also, in this review, our data mining prognostic and mutational analysis, which would provide a brief insight into other cancers where BRK is therapeutically interesting. Following which, known inhibitors of BRK are consolidated, together with a comprehensive overview on the connection between BRK-mediated effects and drug action. Finally, we critically discuss the challenges of inhibitor development, and highlight important considerations for future drug development.

## BRK and Hallmarks of Cancer

### Self-sufficiency in growth signals, evading growth suppressors, and resisting cell death

Cancer cells are errant cells that are able to exploit cell-signaling processes to promote their growth and survival. It has been noted that cancer cells can increase receptor levels, amplify constitutive receptor activation or trigger permanent activation of downstream effectors to promote growth factor independence 13. Desensitizing cells to anti-proliferative signals and growth suppressors deregulates tissue homeostasis and further helps to increase cell survival. Circumventing cell death is another key method for cancer cells to thrive. In this section, we summarize all the known interacting partners and targets of BRK in breast cancer and demonstrate how BRK mediates the three hallmarks of cancer- self-sufficiency in growth signals, evading growth suppressors and resisting cell death. BRK is a convergent point for both upstream growth receptors and downstream effectors, and this extensive influence appears to translate to a broader impact clinically as BRK has also been found to modulate drug sensitivity.

The first report to demonstrate the existence of an interaction between BRK and EGFR also showed that BRK expression could increase the proliferative activity of mammary epithelial cells 14. Subsequently, many studies have reported an association between the two. EGF stimulation was found to lead to an increase in BRK tyrosine phosphorylation 14, 15. Activated BRK was also found to directly phosphorylate tyrosine 845 in the EGFR kinase domain 16. Moreover, BRK is able to further sustain EGFR signaling, through inhibiting ubiquitination of EGFR 16. BRK has also been found to bind to phosphorylate the tyrosine residue on ARAP1, a protein that plays a role in endocytosis 17. BRK phosphorylation of ARAP1 inhibits EGFR internalization and degradation 17. BRK dependent enhancement of EGFR signaling then confers proliferative advantage to breast cancer cells 14, 18. Clinically, the interaction between BRK and EGFR has wide implications, as BRK may potentially be a factor for the low efficacy of anti-EGFR drugs in breast cancer treatment. Indeed, depletion of BRK was found to sensitize cells to Cetuximab 16.

Studies have also shown an association of BRK with human epidermal growth factor receptor 2 (HER2), with co-amplification and co-expression of both proteins in breast cancer cells 19. HER2 has no identified endogenous ligand but heterodimerizes with HER3 and HER4, both of which bind Heregulin. Importantly, Heregulin was found to activate the tyrosine kinase activity of BRK 20. *In vivo*, BRK together with HER2 is also able to increase proliferative capabilities even before tumor development 21. *In vivo* knockdown studies also showed that depletion of BRK and/or HER2 significantly reduces tumor growth 21, 22. The activation by Heregulin on BRK also resulted in activation of extracellular signal regulated kinase 5 (Erk5) and p38 mitogen-activated protein kinases (MAPK) 20. BRK overexpression was found to selectively heighten the Ras/MAPK signaling pathway through sustaining Erk1/2 activation 19, which in turn regulates cell proliferation 23. In a comprehensive study by Lofgren *et al.*, BRK expression was found to promote activation of p38 MAPK *in vitro*, *in vivo*, and in IHC analysis of breast tumor biopsies. Phenotypically, this is exhibited through increased cell survival, and delayed mammary gland involution *in vivo* 24. In this regard, BRK also possesses translational relevance. The same study by Lofgren *et al.* highlighted that mammary epithelial cells expressing BRK appear to promote resistance toward Doxorubicin, as compared to vector control cells 24. Another study also found BRK down-regulation induces apoptosis, via the p38 MAPK pathway. The induction in apoptosis by BRK inhibition in turn abrogated Tamoxifen and Fulvestrant drug resistance in ER+ breast cancer cells 7. BRK down-regulation was even able to eliminate primary tumor growth of drug resistant MCF-7 xenograft 7.

The insulin-like growth factor receptor (IGFR) family has also been implicated with BRK. IGFR signaling pathway chiefly mediates cell survival and prevention of programmed cell death 25. IGF-1R is also proposed as a prognostic breast cancer biomarker as it is found on all breast cancer subtypes to be indicative of poor prognosis 26. Its ligand, IGF-1, has been found to stimulate and increase tyrosine phosphorylation of BRK 27. Interestingly, BRK was found to further enhance IGF-1R signaling. A study ascertained that BRK could modulate IGF-1R phosphorylation by complex formation between BRK and IGF-1R 28. This regulation of IGF-1R signaling in turn results in the promotion of mammary cancer cells' anchorage dependent survival 28. Down-regulation of BRK in MCF-7 breast cancer cells also resulted in decreased IGF-1R autophosphorylation which eventually led to a decrease in extracellular regulated kinase (Erk) and Protein Kinase B (Akt) signaling downstream of IGF-1R 28. Akt is involved in an important pathway to trigger the release of anti-apoptotic signals and thus the prevention of cell death. An *in vitro* study also showed Akt to be a direct substrate of BRK. In HEK293 cells, it was found that BRK binds to Akt through its SH3 and SH2 domains, and directly phosphorylates tyrosine residues 315 and 326 leading to Akt activation 29. BRK was also found to sensitize cells to EGF activation of Akt 29. Again, BRK oncogenic properties may potentially be extended to translational applications. Ectopic expression of BRK increased levels of Akt and Erk phosphorylation, and decreased the efficacy of Cetuximab wherein higher doses of Cetuximab was required to achieve the same level of growth inhibition as compared to control breast cancer cells 16. It has also been found that BRK inhibition can promote apoptosis in Lapatinib-resistant HER2 positive breast cancer cells by inducing Bim 30.

Besides its regulation by upstream EGF and IGF, BRK appears to also be a target of glucocorticoid receptor (GR). GR, in a complex with co-activator PELP1 and hypoxia-inducible factors (HIF), was found to bind to BRK promoter region and increase BRK expression 31. BRK upregulation then in turn promoted cell survival *in vitro*. Therefore, targeting GR may be a good way to alter BRK expression, and BRK may also be used as an important biomarker of potentially activated GR in tumors. It is important to note that while the phospho-GR/HIF/PELP1 complex may induce cell survival, the same study found that ectopic expression of Brk can bypass this requirement and in itself promote cell survival, perhaps in part due to feed-forward signaling through Brk-induced activation of p38 MAPK that was aforementioned.

BRK have also been shown to mediate signal transducers and activators of transcription 3 (STAT3) activation 32. STAT3 is an important transcription factor that can be activated to boost cell proliferation and survival 33-35. The modulation of STAT3 is executed through BRK's interaction with signal transducing activator protein 2 (STAP-2), which subsequently interacts with STAT3 36, 37. STAP-2 was one of the first substrates of BRK to be uncovered and it is phosphorylated on tyrosine-250 by BRK 36. BRK, STAP-2 or STAT3 knockdown all gave similar degrees of reduction in T47D breast cancer cell proliferation 37. Intriguingly, Liu *et al.* discovered that STAT3 is directly phosphorylated by BRK on tyrosine-705 in a dose-dependent manner 38. A possible mechanism of action (MOA) to explain the findings gathered here so far is that BRK, STAP-2, and STAT3 form a complex in cells where BRK directly phosphorylates both STAP-2 and STAT3. This result in the activation of STAP-2, which binds to STAT3 to further enhance the transcriptional activity of STAT3 37, 39, 40.

Besides STAT3, STAT5b is another molecule that physically interacts with STAP-2. While STAT3 binds to STAP-2 through its C-terminal YXXQ motif, STAT5b and STAP-2 interact through their pleckstrin homology and SH2-like domains 39. BRK was found to mediate STAT5b phosphorylation at tyrosine-699, the activating residue of STAT5b 41. The same group of researchers also showed that in breast cancer cell lines expressing BRK, siRNA-mediated knockdown of BRK or STAT5b reduced DNA synthesis but there was no further decrease for the double knockdowns 41.

It has been found that BRK can aid evasion of growth suppressor retinoblastoma protein (Rb) gatekeeping effects through the upregulation of cyclin D and E, pushing cells towards synthesis and proliferation. Rb, an important tumor suppressor, inhibits cell growth by serving as a gatekeeper between the G1 to S phase of the cell cycle. Its phosphorylation is mediated by cyclin E-cyclin dependent kinase 2 (CDK2) and cyclin D-CDK4/6 complexes. Phosphorylation of Rb inactivates its growth inhibitory functions, pushing cells to enter the DNA synthesis stage 42. It has been found that BRK overexpression promotes HER2-induced cell proliferation via increasing activation of the cyclin E-CDK2 complex 19. Conversely, BRK knockdown studies resulted in a strong cyclin E level reduction and attenuated cyclin D levels 43, 44.

p27 is another growth suppressor that can inhibit the proliferation that results from the phosphorylation of pRB. BRK can down-regulate p27 by inhibiting the nuclear localization of its transcription factor forkhead box protein O (FOXO), thereby antagonizing its transcriptional activity 45. It was also found that when BRK is expressed in HER2-induced cells, levels of p27 dipped at a faster rate compared to control cells. This suggests that BRK together with HER2 is able to promote cell cycle progression through downregulating the growth suppressor p27 19. In another study, similar results were also reported where overexpression of BRK decreased both p27 protein and mRNA levels. Knockdown studies produced similar results where an increase in the levels of p27 were observed following BRK silencing 45.

Taken together, BRK contributes extensively to cancer development by amplifying effects of various upstream transmembrane receptor tyrosine kinases and causing activation of downstream effectors to confer cell viability. It has a wide berth of influence on both oncoproteins and tumor suppressors alike.

### Activating invasion and metastasis and inducing angiogenesis

As cancer develops toward increased malignancy, cells will detach, migrate, invade surrounding tissues and eventually establish a new colony. The formation of new blood vessels for the providence of nutrients and oxygen is also important for cancer dissemination. Particularly, BRK is heavily implicated in the process of epithelial-mesenchymal transition (EMT) and angiogenesis. In 2004, BRK was first shown to promote EGF-induced cell migration 15. Chen *et al.* reported that EGF stimulation activates the catalytic activity of BRK, which in turn phosphorylates paxillin to promote the activation of Rac1, a guanosine triphosphate hydrolase (GTPase). Paxillin is an extracellular matrix tethering protein that localizes to focal adhesions and regulates interactions between actin cytoskeleton and the ECM 46, 47. BRK translocate to membrane ruffles and colocalizes with paxillin during cell migration 48, 49. The EGF pathway also stimulates BRK's phosphorylation of paxillin to promote migratory and invasive characteristics in breast cancer cells. BRK has been reported to directly phosphorylate paxillin at tyrosines-31/118 and promote migration via activation of Rac1 GTPase 15.

In a follow-up study, the research group also identified BRK's role in phosphorylating p190RhoGAP-A (p190-A) at tyrosine-1105 upon EGF stimulation 50. Phosphorylated p190-A then associates with p120RasGAP (p120) to inhibit the latter's activity, consequently leading to inhibition of RhoA and activation of the Ras oncogene to promote migration and invasion 50. In breast cancer cell lines, the results were confirmed by the observation that RhoA and Ras regulation was lost after severing the association between p190-A and p120 50. Derry *et al.* also reported that by phosphorylating p190RhoGAP, BRK regulates Rho and Ras to promote breast carcinoma growth, migration, and invasion 51.

In addition, *in vitro* work has demonstrated that focal adhesion kinase (FAK) is another direct BRK substrate 52. *In vivo*, BRK knockdown prevented the phosphorylation of FAK at tyrosine residue 576/577, resulting in fewer metastases 21. The same report also identifies breast cancer anti-estrogen resistance protein 1 (BCAR1) to be a substrate of BRK, where *in vivo* knockdown of BRK disrupts phosphorylation of BCAR1 tyrosine residue 165, preventing its activation. Phosphorylation of BCAR1 promotes cell migration and invasion 53. Other than FAK and BCAR1, SMAD4 appears to be another target phosphorylated by BRK 54. Importantly, BRK's phosphorylation of SMAD4 targets it for ubiquitination and degradation, which in turn leads to a repression of its downstream target, the tumor suppressor fyn-related kinase (FRK), which is involved in EMT processes. Constitutively active BRK therefore, was found to promote levels of EMT transcription factors SNAIL and SLUG in breast cancer cells 54. This concurs with another study showing that BRK down regulation affects E-cadherin levels through compromising the posttranscriptional stability of transcriptional repressor SNAIL 5.

Besides these effectors, Irie's group also reported that in DOV-13 ovarian cancer cells and in breast cancer cells, BRK appears to have a positive role in affecting IGF-1 induced anoikis, which is a form of programmed cell death for anchorage dependent cells when they detach from the extracellular matrix (ECM) 28. Tumor cells with anoikis resistance are able to survive after detachment from the primary site and thus metastasize.

BRK is also a key mediator in hypoxia-induced breast cancer progression. Interestingly, it should first be noted that there exist disagreements regarding the mechanism of action of this hypoxia-mediated BRK induction. In 2014, Pires *et al.* finds BRK to be stabilized in hypoxic condition in a HIF-1α independent manner in breast cancer cells 55. The team has also refuted the hypothesis that BRK may affect HIF-1α activity. However, Regan Anderson *et al.* observed that BRK is a direct transcriptional target of the HIF-1α and that BRK's promoter consists of HIF-responsive elements (HRE), which actively recruits HIF-1α particularly in conditions of hypoxia 56. In tumors with HIF-1α knocked out, protein levels of Sik (the mouse homologue of BRK) were significantly reduced as compared to tumors expressing wildtype HIF-1α 56. This is in agreement with another finding in 2016 that the GR/HIF/PELP1 complex binds to the BRK promoter region and increase BRK expression 31. Nevertheless, despite the conflict regarding the involvement of HIF-1α, both studies by Regan Anderson and Pires corroborated that conditions of hypoxia can trigger the induction of BRK. Additionally, Pire *et al*'s study highlighted that hypoxia-mediated BRK induction promotes MDA-MB-231 cell growth in monolayer as well as 3D mammosphere growth 55. Regan Anderson *et al.* showed that *in vivo*, 40% of HIF-1α and HIF-2α knockout mice transfected with cells overexpressing constitutively active BRK displayed macrometastasis of the lymph node as compared to none in the group with null BRK, or HIF-1α and HIF-2α knockouts 56. The results highlight that BRK is a major mediator of hypoxia-induced metastasis.

A study has found that osteopontin triggers vascular endothelial growth factor (VEGF)-dependent tumor progression and angiogenesis by activating BRK/nuclear factor-inducing kinase/nuclear factor-kappaB/activating transcription factor-4 signaling cascades through autocrine and paracrine mechanisms 57. In addition, BRK is found to be co-expressed with HIF-1α, a key regulator of pro-angiogenic VEGF 56, 58. It was found that in some cancers, overexpression of HIF-1α is associated with VEGF expression and vascularization 58. Overall, BRK is an important oncoprotein that promotes cell migration and invasion.

### Reprogramming of energy metabolism and evasion of immune destruction

Eleven years after the first six foundational hallmarks of cancer was described, Hanahan and Weinberg added two more emerging hallmarks of cancer. The first involves protection against attack by immune cells so as to sustain uncontrolled cell growth and the second is the major reprogramming of energy metabolism 13.

It has been reported that BRK is persistently activated in transformed T and B cells population as well as Epstein-Barr virus (EBV) transformed cells 59. The same study showed that *in vivo*, BRK has the oncogenic potential to induce malignant transformation similar in nature to that which is seen in lymphoid malignancies. Taken together, BRK appears to have a strong association with T and B cells and their transformation. However, in the context of breast cancer, BRK's role in immune evasion is still largely unexplored.

Reprogramming of energy metabolism, the second emerging hallmark of cancer, can occur through glycolysis up-regulation 13. In prolonged hypoxic conditions, HIF-1α plays a central role for activating transporters and enzymes for glycolytic fueling, increasing cell survival and enhancing tumorigenesis 60. While there has been no report measuring the direct relation between BRK and glycolysis, it has been reported that BRK is co-expressed with HIF-1α and HIF-2α, which are important transcription factors that upregulate glycolysis 56, 60. BRK levels are also elevated in stressed cellular environment with oxygen deprivation 56.

**Figure 2:**
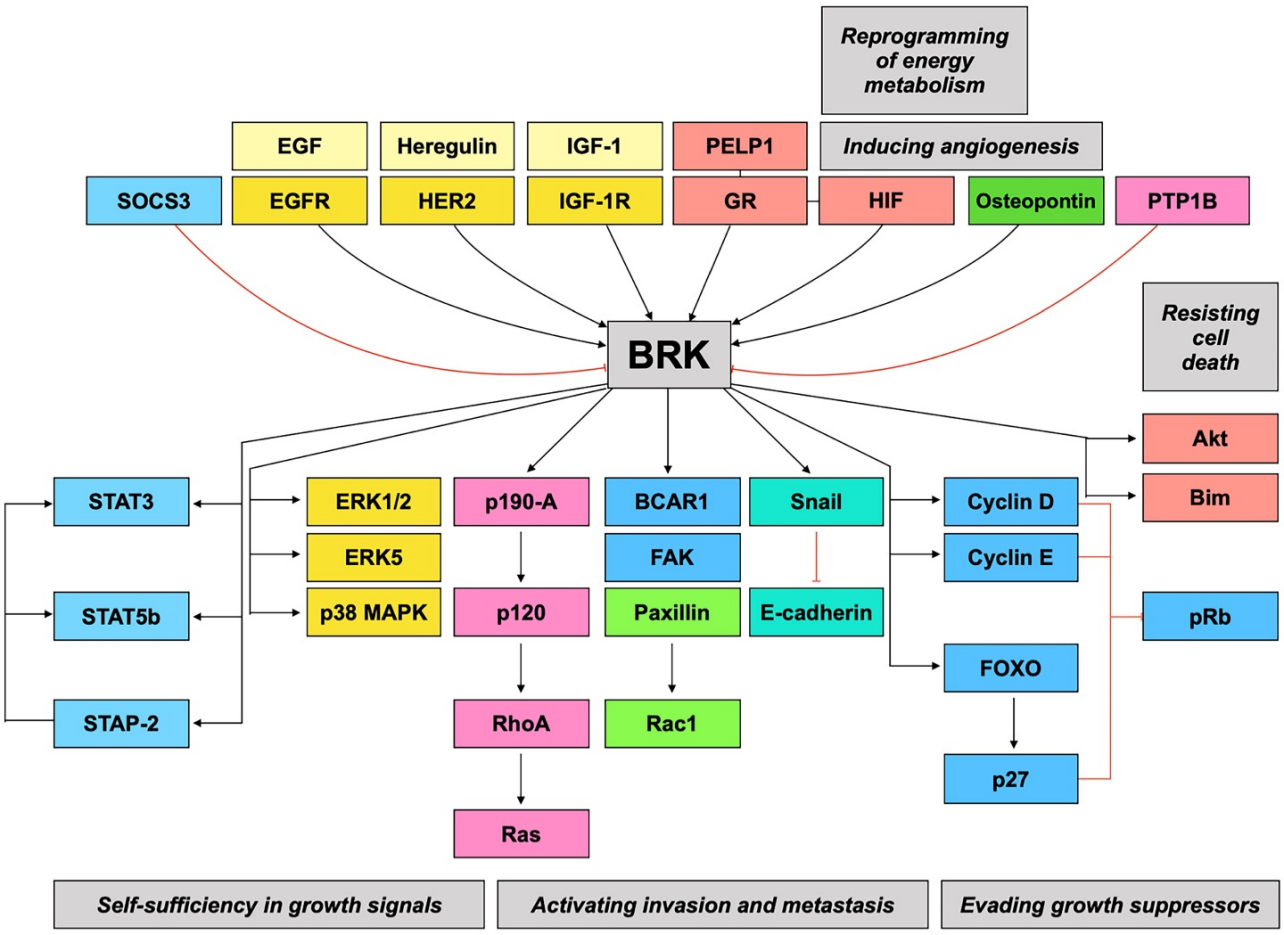
** BRK signaling pathways.** BRK is the converging point of a variety of signaling pathways that result in modulation of different hallmarks of cancer.

Taken together, BRK is implicated in numerous signaling pathways and may therefore be a highly relevant therapeutic target. **Figure 2** gives a summary of the preceding discussions, linking BRK's molecular targets and their subsequent downstream phenotypes.

## Therapeutic Significance

**Figure 3:**
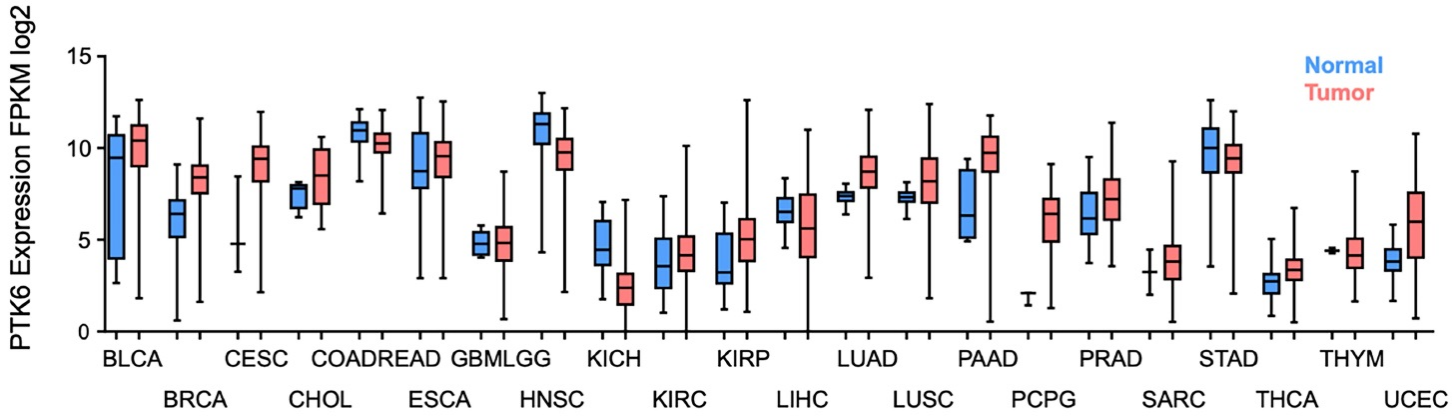
** Clinical characterization of BRK expression level in cancer.** Boxplot of *PTK6* expression (*y*-axis) in normal (blue) and tumor (red) of the TCGA carcinoma cohorts. (BLCA, bladder urothelial carcinoma; BRCA, breast invasive carcinoma; CESC, cervical and endocervical cancers; CHOL, cholangiocarcinoma; COADREAD, colorectal carcinoma; ESCA, esophageal carcinoma; GBMLGG, glioblastoma and low grade glioma; HNSC, head and neck squamous cell carcinoma; KICH, kidney chromophobe; KIRC, kidney renal clear cell carcinoma; KIRP, kidney renal papillary cell carcinoma; LIHC, liver hepatocellular carcinoma; LUAD, lung adenocarcinoma; LUSC, lung squamous cell carcinoma; PAAD, pancreatic adenocarcinoma; PCPG, pheochromocytoma and paraganglioma; PRAD, prostate adenocarcinoma; SARC, sarcoma; STAD, stomach adenocarcinoma; THCA, thyroid carcinoma; THYM, thymoma; and UCEC, uterine corpus endometrial carcinoma).

Considering all the evidence regarding BRK's oncogenic role in contributing to the hallmarks of cancer (**Figure 2**), it becomes pertinent to discover BRK's potential as a therapeutic target for the development of novel treatments for cancer. Using data curated from the TCGA dataset, there appears to be distinctively elevated levels of BRK in a high proportion of cancer tissues as compared to normal tissue (**Figure 3**) 61. This overexpression in turn influence patient prognosis. Patient tissue analysis of bladder 62, cervical 4, prostate 63, and non-small cell lung cancer 64 showed that higher expression of BRK is associated with poorer overall survival. Particularly, in cervical 4, thyroid 65, and bladder cancer 62, BRK expression levels are also associated with tumor grade and severity. Interestingly in breast cancer, Aubele *et al.* highlighted in year 2007 and 2008 that BRK appears to be a positive indicator of disease-free survival 66, 67. Ten years later in 2017, Ito *et al.* highlighted based on TCGA analysis of a larger cohort, that BRK expression is associated with poor overall survival 7. The contrasting outcome could be attributed to a smaller cohort size and the different composition in tumor cohort in Aubele's group. Nevertheless, both authors concurred that BRK levels are associated with increased carcinogenic markers (Sam68, MAPK), and that BRK may be a potential target for targeted therapy. BRK protein expression levels also correlated with earlier recurrence and increased metastasis in prostate cancer 63. Similarly in breast cancer, higher BRK expression correlated with reduced metastasis-free survival 55. We have also observed that BRK levels are notably higher in cholangiocarcinoma and pancreatic adenocarcinoma (**Figure 3**). In these cancers, BRK have been found to play a role in cell proliferation, migration, and invasion 68, 69. Taken together, BRK's overexpression and its mediation on the various hallmarks of cancer hint at the oncogenic nature of BRK. BRK's oncogenicity is further highlighted by the increased cancer severity and poorer overall survival that is associated with increased BRK expression. BRK therefore proves to be a promising therapeutic target and may be a viable biomarker in cancer. Finally, the role of BRK in paraganglioma, kidney renal papillary cell carcinoma, and uterine corpus endometrial carcinoma remains to be elucidated. However, according to our data analysis, it appears that BRK is overexpressed in these cancers, and it may be clinically useful to understand the significance of BRK overexpression in these cancers.

In addition, we have also provided in this review BRK mutation profiles in various cancers (**Table 1**) 61. Importantly, pathogenicity prediction from three methods (Mutation Assessor 70, SIFT 71, and MetaLR 72) which predict the effect of change on protein function is included. These scoring methods generally make use of allele frequency information or sequence homology to predict whether the amino acid substitution will affect protein function and result in a phenotypic effect. Out of the 13-missense mutation of BRK with annotation, 11 were predicted to be deleterious and only 2 were predicted to be tolerated (**Table 1**). However, further mutagenesis work needs to be performed to ascertain the true effect of the mutation on the BRK protein.

BRK mutations mostly fall on the kinase domain (19/32). Mutations on the kinase domain may be the most significant, as BRK has been found to directly phosphorylate and activate various downstream targets such as Akt, EGFR, STAT3 16, 29, 38. The second most common sites of BRK mutation were the SH2 and SH3 domains (5/32 for both). These two domains have been found to be important for protein-protein interactions. For instance, BRK was found to interact with Akt via its SH2 and SH3 domains 29. The SH3 domain is also recognized for its substrate recognition function 73. A mutation in the SH3 domain may therefore result in erroneous signaling or off-target activation of various oncoproteins. Additionally, it has been reported that the SH2 and SH3 domains are likely to have a role in regulating BRK kinase activity 74. SH2 was reported to be involved in the negative regulation of the kinase activity through the tyrosine 447-SH2 interaction 74, 75. Indeed, mutation of arginine at site 131 in the BRK SH2 domain was found to result in increased BRK autophosphorylation and increased BRK activity 76. Unlike SH2, SH3 interacts with the linker region to regulate kinase activity. A site-directed mutation of tryptophan at position 44 interrupts the SH3-linker interaction and significantly enhanced kinase activity of BRK in HEK293 cells 74. Finally, the BRK linker-kinase interaction has an essentially positive role in regulating catalytic activity; introduction of a substitution mutation at position 184 abrogated BRK autophosphorylation and inhibited downstream cell proliferation of HEK293 cells 77. Interestingly, this is in contrast to the Src family kinase, where the linker-kinase interaction inhibited kinase ability. Taken together, the SH2, SH3 and linker domains appear to indirectly regulate kinase activity. A better understanding of the list of mutations (**Table 1**) is therefore warranted as a mutation in either domain may promote BRK activity and downstream cancer progression.

Other than the mutation profiles as summarized in **Table 1**, a study by Schmandt *et al*. also showed through a fluorescent *in situ* hybridization (FISH) experiment, that *PTK6* gene is amplified in ovarian cancer 3. *PTK6* gene was also discovered to be amplified in prostate cancer 78. The mutation information highlights the need for BRK inhibitors, which could potentially alleviate the conditions of cancer patients with aberrant BRK levels and activity.

Building on the understanding of BRK's oncogenic role in breast cancer, and with it the poorer treatment prognosis, we discuss in the next section current known inhibitors of BRK in breast cancer. We seek to understand how these inhibitors can attenuate BRK-mediated cancer progression, and to also learn from the success and challenges of these developments.

## BRK Inhibitors

Biological inhibitors are cellular compounds that target BRK and/or its associated pathways. The suppressor of cytokine signaling 3 (SOCS3) protein was observed to be a negative regulator of BRK 79. Conventionally, SOCS3 has been studied as a feedback inhibitor regulating the JAK-STAT pathway through both ubiquitin-mediated proteasome degradation and non-competitive inhibition. BRK, as an activator of STAT3, was also found to be the target of SOCS3 negative modulations. This is mediated via SOCS3 interaction with BRK tyrosine kinase domain. Its inhibition of BRK in turn prevented downstream phosphorylation of STAT3, and reduced proliferation of MDA-MB-231 and T-47D breast cancer cell lines. While only *in vitro* research has been performed, this is the first evidence of a biologically available BRK inhibitor, and SOCS3 holds potential to be exploited as a tumor suppressor to block BRK-mediated cancer progression.

Besides the above biological inhibitor of BRK, an increasing number of chemical inhibitors have also been studied. Triterpene sipholenols, isolated from the Red Sea sponge *Callyspongia siphonella*, and their semisynthetic derivatives have been discovered to be selective BRK inhibitors, and are capable of inhibiting BRK phosphorylation in a dose-dependent manner 77. The most potent triterpene sipholenol BRK inhibitors were identified to be 4β-O-benzyl sipholenol A and 4β-O-benzyl-19,20-anhydrosipholenol A 78. It was acknowledged that BRK promotes cell dissemination in breast cancer cells. The authors therefore first performed an *in vitro* assay on the MDA-MB-231 breast cancer cell line to evaluate their anti-migratory activities. It was observed that the effectiveness of analogues in inhibiting BRK phosphorylation paralleled their anti-migratory ability 80. Importantly, none of these compounds exhibited cytotoxicity in normal breast cell line.

Oleanolic Acid 81, extracted from Terminalia bentzoe L. leaves, is another triterpene whose semisynthetic derivatives have been optimized in anti-migration, anti-proliferation, and anti-invasion effects on the breast cancer cell line MDA-MB-231, and was further shown to induce apoptosis in four breast cancer cell lines: MDA-MB-231, MCF-7, BT-474, and T-47D. These effects were proposed to be in part due to the derivatives' ability to inhibit phosphorylation of BRK, along with Paxillin and Rac1, and in part due to upregulation of FASL, leading to activation of RIP, BID, and various caspases, and eventually to the proteolytic cleavage of PARP-1 80.

Similar to the semisynthetic derivatives from oleanolic acid, (Z)-5-((4′-Fluorobiphenyl-10-yl)methylene)imidazolidine-2,4-dione was also found to significantly decrease phosphorylation of BRK, paxillin, and Rac1, with little effect on their total levels 82. This compound is the most active derivative of Phenylmethylene hydantoins, a natural compound that is isolated from marine sponge *Hemimycale Arabica*. Additionally, the analogues displayed anti-proliferative and anti-migratory effects in breast cancer cell line MDA-MB-231, while being non-toxic to normal mammary epithelial cells at the concentrations tested on the cell line MCF-10A.

Interestingly, heat shock protein 90 (Hsp90) inhibitors, such as geldanamycin, can also be considered as a therapeutic agent to indirectly inhibit BRK 83. Proteosomal degradation of BRK is ubiquitin-mediated, and this process is impeded by increased protein stability rendered through BRK-Hsp90 interaction. Geldanamycin, which prevents this heteroprotein complex formation, reduces BRK levels in a time-dependent manner in breast cancer cell lines T-47D and BT-474 and decreases phosphorylation of BRK substrates. It was also observed that the attenuation of BRK phosphorylation in turn inhibits BRK-mediated activation of its direct targets, STAT3, SAM68, and Paxillin.

Among novel chemical compounds generated to specifically inhibit BRK, Imidazo[1,2-a]pyrazin-8-amines was the first to be synthesized. Docking studies find this compound to be capable of interacting with BRK's ATP-binding pocket and thereby inhibit it 84. Biochemical studies optimized a subclass of analogues (21a and 21d) to be the most selective against BRK. Importantly, cellular activity showed that 21a and 21d were effective in attenuating the phosphorylation of SAM68, a substrate of BRK 84. Unfortunately, this compound was revisited in 2018, and a kinase panel screening revealed that the analogues unselectively target 6% of the kinases in the panel of 320 kinases 85.

4-anilino α-carbolines are another class of compounds that have been studied as BRK inhibitors, of which 4-(m-hydroxyaniline)-α-carboline (4f) was found to be the most potent 86. It was predicted that this inhibition occurs through interaction with BRK's ATP-binding pocket. Based on BRK's known association with HER2, the study first determined if 4-anilino α-carbolines analogues are able to reduce receptor-mediated proliferative effect. Indeed, the analogues, particularly 4f, was found to be successful in limiting proliferation of MCF7, HS-578/T, and BT-549 breast tumor cancer cell lines, with correlation observed between effectiveness of BRK inhibition and anti-proliferative effects of these compounds. In a follow-up study, these compounds also significantly reduced the phosphorylation of STAT3, a direct BRK target, and were found to modestly induce cell death of non-adherent breast cancer cells 87.

(E)-5-(benzylideneamino)-1H-benzo[d]imidazol-2 (3H)-one derivatives showed effectiveness in inhibiting phosphorylation of BRK, with at least 20-fold selectivity over similar non-receptor tyrosine kinases, Src, Fyn, Bmx, and EGFR 88. Two of the most potent compounds (compounds 20 and 21) also displayed low cytotoxicity in normal human foreskin fibroblast. A preliminary proof of concept for compounds 20 and 21 is then established through *in vitro* testing, where they effectively decreased the phosphorylation of Paxillin and STAT3.

XMU-MP-2 is another chemical inhibitor that was designed to specifically inhibit the kinase activity of BRK 89. The specificity of XMU-MP-2 is ascertained through the measuring of Y342 phosphorylation, as well as the measurement of STAT3 and STAT5 phosphorylation, both of which have been found to be direct BRK targets. Importantly, XMU-MP-2 has been described to be “on-target” to BRK, as the drug MOA is largely in concordance to the targeting of BRK-mediated effect. Particularly, XMU-MP-2 also decreases Erk1/2 and Akt phosphorylation. This novel inhibitor has also been shown to block breast cancer cells proliferation and induce apoptosis *in vivo* and *in vitro*. Additionally, XMU-MP-2 may possess an added advantage of being employed in combination therapy. It displayed strong synergy with HER2 and ER inhibitors such as CP-724714 and Tamoxifen.

In 2017, through an *in vitro* kinase assay, it was discovered that Lck inhibitors Pyrazolopyrimidine PP1 and PP2 were able to inhibit the catalytic activity of BRK. PP1 and 2 effectively suppressed the phosphorylation of BRK substrate, STAT3, in HEK 293 cells. The authors also showed that PP1/2 inhibited BRK-dependent proliferation of T-47D breast cancer cells, which was consistent with the effect of BRK knockdown 90.

Finally, in 2018, PF-6683324 and PF-6689840 were designed specifically to bind to unphosphorylated BRK 85. Based on a kinase panel screening, these compounds appear to be selective toward BRK. However, the effect of these compounds on BRK kinase activity *in vitro* was not ascertained. Moreover, off-target effects were also observed *in vitro*. Treatment with BRK kinase inhibitor PF-6689840 and structural analogue PF-6737007 that lacks BRK activity yielded similarly poor cell growth inhibition of MDA-MB-231 cells 85. Interestingly, after rigorous experimentation, Qiu *et al.* concluded that the kinase specific inhibitors did not yield any anticancer efficacy 85. This suggests that the kinase independent functions of BRK may play an important role in oncogenesis. Most of the inhibitors that have been discussed thus far targets BRK kinase activity. This may be a crucial factor impeding the further development of BRK inhibitors.

Beyond these novel and newly synthesized compounds, two drugs currently in the market also appear to target BRK. Firstly, Dasatinib was found to bind to the front, gate and subpockets of BRK's ATP binding pocket , and exhibited high potency with an IC_50_ value of 7nM 91. Dasatinib is an FDA approved drug for the treatment of chronic myeloid leukemia. However, it has exhibited efficacy in halting EMT in the breast cancer model 92. It was also found to improve the efficacy of Paclitaxel in breast cancer when used in combination 92. Unfortunately, the MOA of Dasatinib against BRK has not been explored. The *in vitro* and *in vivo* efficacy of Dasatinib is also unknown. More importantly, it is important to note that Dasatinib is also highly selective against other kinases such as Bone Marrow kinase on chromosome X (BMX), and Bruton Tyrosine Kinase (BTK) 91, and such blanket inhibitions are thought to contribute to the off-target effects observed in the clinical settings.

Recently, Verumafenib was also shown to also selectively inhibit BRK through binding to its active site 93. The inhibition of BRK resulted in a decrease in oncogenic properties in prostate cancer, mainly through cell growth and EMT inhibition. Veuamafenib targeting of BRK also in turn reduced tumor burden *in vivo*. While, this drug inhibition of BRK has only been exhibited in the prostate cancer model, it presents a drug MOA that is consistent with the targeting of BRK. Its effect against BRK in the breast cancer model remains to be elucidated.

Overall, there has been much progress to identify novel drugs targeting BRK. This development is crucial as there are compelling evidence to suggest BRK oncogenicity, yet there are no direct-targeted therapeutics available. Generally, we do observe that most of these drug compounds effectively reduced BRK-mediated oncogenicity. However, the analysis of these BRK inhibitors also revealed challenges in drug design. There remains much potential in the exploration of therapeutics against BRK. Considering current difficulties in therapeutics design, in the next section, we discuss our perspectives on how BRK should be attacked for a viable therapy.

## Future Perspectives

### BRK and hallmarks of cancer

Prior to the discussion regarding challenges in therapeutics design, we first discuss the knowledge gap regarding BRK's contribution toward cancer progression. Carcinogenesis is a multistep and complicated process as there could be crosstalk between different molecules (i.e. BRK's effects on Erk1/2 and RhoA through different pathways eventually activate Ras to cause metastasis) or a certain oncogenic process may trigger multiple capabilities (i.e. Ras not only confers self-sufficiency in growth signals, but also activates invasion and metastasis) 13. Therefore, it is important for us to have a complete understanding on the different factors regulated by BRK, to better understand its role in promoting tumorigenesis. While BRK contribution toward cancer progression in breast cancer has been largely demonstrated, there are still uncharted angles to explore vis-a-vis BRK's association with other hallmarks of cancer.

Genome instability and mutation is recognized as an important enabling characteristic for cancer development 13. The DNA damage response (DDR) angle is also particularly relevant for breast cancer. It has been observed that while BRCA mutation affects the DDR pathway and should heighten the risk for all cancers, BRCA mutation typically results in cancer with breast or ovarian origin. It has been suggested that estrogen regulation may increase double stranded break, which may therefore explain tissue specificity 94. However, BRK's influence on the DDR pathway in breast cancer remains largely unexplored. Perhaps, this is where linking BRK to DDR pathways would be an emerging field. In 2015, Bourton *et al.* showed that in MDA-MB-157 triple negative breast cancer cell line, BRK overexpression significantly confers resistance toward cellular radiation, thereby resulting in increased cell survival upon gamma radiation, as compared to cells with BRK kinase mutant or empty vector 95. Interestingly, this phenotype was not observed in MDA-MB-468 breast cancer cell line. Little has been published regarding BRK association with DDR in breast cancer. It may therefore be worthwhile to further dissect the mechanism of action of BRK in DDR pathway or DNA damage repair systems, and to understand why these effects are seen in certain cell lines and not others. For a start, published literature show that BRK could confer resistance to DNA damage via its modulation of the EGFR signaling pathway 95. In other cancer models, there is also little information relating BRK to the DDR pathway. It was reported that siRNA depletion of BRK regulates DDR through increased p21 expression in wild type p53 colon cancer 96, while PTK6 overexpression enhanced gemcitabine-induced DNA damage in pancreatic cancer cells 97.

In another instance, the hallmarks that have been discussed in the earlier section chiefly describe aberrant cell growth resulting from cell signaling. However, intrinsically, cellular replicative capacity is kept in check by telomeres, which serves as a way to hinder expansive tumor growth. When telomeres are dysfunctional, the extent of tumorigenesis is greatly accelerated, with endless replicative immortality conferred to already rapidly growing and death-resistant cells. When comparing *PTK6* and its mouse homologue *Sik,* it was found that the coding region of both genes are located near the telomere 98. One might therefore speculate that mutational alterations in BRK could result in telomeric aberrations as well. The association between BRK and the hallmark “limitless replicative potential” is therefore another interesting area that has not been explored, but may prove to be highly relevant in further understanding cancer development.

### BRK inhibitors development

In the earlier section “BRK inhibitors”, we summarized a series of inhibitors that have been developed against BRK. Unfortunately, therapeutics design has been riddled with various challenges, and despite more than 5 years since the publication of the first inhibitor, there are currently no drugs that have progressed to clinical testing. It is therefore important for us to critically consider these challenges to improve future BRK drug design.

Generally, poor selectivity appears to be a shortfall among many of the BRK inhibitors. Particularly, imidazo[1,2-a]pyrazin-8-amines have shown modest cellular activity in inhibiting the phosphorylation of SAM68, a BRK substrate, but was later refuted due to its poor selectivity. Additionally, there are also inhibitors with unknown cytotoxicity results 84, 87, 90.

One of the most important observations would be that current inhibitors primarily work through blocking BRK phosphorylation, leaving the SH2 and SH3 domains free to interact with other substrates. Research has shown that catalytically inactive form of BRK is still able to enable proliferation and motility of breast cancer cells 76, 99, 100. In T47D cells, kinase dead BRK boosted cell proliferation as compared to control cells transfected with empty vector, highlighting a kinase independent role for BRK in regulating breast cancer cell growth 99. Subsequently, research by Castro and Lange also revealed that both wildtype and catalytically inactive BRK were able to activate Erk5 and promote HGF-induced cell migration 48. There appears to be a lack of a strict requirement for the BRK kinase activity in certain hallmarks of cancer, such as in cell proliferation and migration. Having free SH2 and SH3 domains may still potentially promote cancer development. This argument is especially evident in the latest BRK inhibitor research. Both the newly developed BRK kinase inhibitor as well as its analogue (that does not inhibit BRK kinase activity) moderately inhibited cell growth in breast cancer cell lines MDA-MB-231 and MDA-MB-453 with similar potency, suggesting again that the inhibition of tumor cell growth may be independent of BRK kinase activity. Taken together, there is strong evidence suggesting the kinase independent function of BRK in oncogenesis. The fervent development of BRK kinase specific inhibitors may need to be reconsidered in light of these new findings.

Nevertheless, it appears that cell survival mechanisms may still require BRK kinase activity. As mentioned previously, it was discovered that BRK directly phosphorylates Akt on tyrosine resides 315 and 326 29. This phosphorylation was achieved by both wild-type BRK and constitutively active BRK. However, kinase mutant BRK failed to induce the phosphorylation of Akt 101. Evidently, BRK kinase functions as well as its SH2 and SH3 domains are critical in promoting cancer development. Therefore, an inhibitor that is singularly inhibiting a particular domain may be ineffective. This may perhaps be a crucial factor impeding progress in drug development despite the growing understanding of BRK's role in carcinogenesis. Moving forward, it is imperative that a more extensive understanding regarding the role of each BRK domain is achieved, before diving into inhibitor development.

## Conclusions

Consolidating research about BRK helps to identify knowledge gaps and also isolate targetable pathways to curb tumorigenesis. With the oncogenic role of BRK reviewed here, the pressing need to translate known or new BRK inhibitors to benefit patients is highlighted. All known BRK inhibitors have been critically consolidated in this review. An understanding of the many complex functions of BRK in cancer, together with the understanding of the successes and challenges in BRK inhibitor development, could help in the development of better drug design and cancer specific therapy.

## Figures and Tables

**Table 1:** BRK mutation profile in various cancers

Disease	Amino Acid Change	Mutation Type	Mutation Domain	Predictions		
				Mutation Assessor	SIFT	MetaLR
BLCA	p.G54D	Missense_Mutation	SH3	M	T	T
	p.L284L	Silent	Kinase	.	.	.
BRCA	p.L248	Silent	Kinase	.	.	.
	p.A252	Silent	Kinase	.	.	.
CESC	p.F434	Silent	Kinase	.	.	.
	p.L276	Silent	Kinase	.	.	.
GBMLGG	p.H8Q	Missense_Mutation	Linker	L	T	T
LIHC	p.S305X	Nonsense_Mutation	Kinase	.	.	.
LUAD	p.D5Y	Missense_Mutation	Linker	L	D	T
	p.Q230Q	Silent	Kinase	.	.	.
LUSC	p.R131P	Missense_Mutation	SH2	.	.	D
OV	p.N317S	Missense_Mutation	Kinase	M	.	D
PAAD	p.R105R	Silent	SH2	.	.	.
	p.F206F	Silent	Kinase	.	.	.
	p.V115V	Silent	SH2	.	.	.
SKCM	p.R316R	Silent	Kinase	.	.	.
	p.D24N	Missense_Mutation	SH3	L	T	T
	p.P389L	Missense_Mutation	Kinase	M	.	D
	p.P356F	Missense_Mutation	Kinase	.	.	.
	p.V216M	Missense_Mutation	Kinase	M	.	D
	p.R195R	Silent	Kinase	.	.	.
	p.G60V	Missense_Mutation	SH3	H	D	D
	p.V37V	Silent	SH3	.	.	.
	p.A53V	Missense_Mutation	SH3	L	D	T
	p.R186R	Silent	Linker	.	.	.
	p.I247I	Silent	Kinase	.	.	.
STAD	p.N323N	Silent	Kinase	.	.	.
	p.F206F	Silent	Kinase	.	.	.
	p.Y251H	Missense_Mutation	Kinase	L	.	D
	p.P169P	Silent	SH2	.	.	.
	p.W130C	Missense_Mutation	SH2	.	.	D
	p.A238D	Missense_Mutation	Kinase	L	.	D

T = Tolerated; D = Deleterious/ Damaging; L = Low risk; M = Moderate risk; H = High risk; BLCA, bladder urothelial carcinoma; BRCA, breast invasive carcinoma; CESC, cervical and endocervical cancers; GBMLGG, glioblastoma and low grade glioma; LIHC, liver hepatocellular carcinoma; LUAD, lung adenocarcinoma; LUSC, lung squamous cell carcinoma; OV, ovarian serous cystadenocarcinoma; PAAD, pancreatic adenocarcinoma; SKCM, Skin Cutaneous Melanoma; STAD, stomach adenocarcinoma.
